# Characteristics of Vaccine-Hesitant and Nonhesitant Parents in Italy: A Cross-Sectional Study

**DOI:** 10.1097/NNR.0000000000000890

**Published:** 2026-02-09

**Authors:** Camilla Elena Magi, Andrea Poliani, Alessia Campoli, Ilaria Marcomini, Giulia Villa, Stefano Bambi, Laura Rasero, Paolo Iovino, Yari Longobucco

**Affiliations:** Camilla Elena Magi, Stefano Bambi, Laura Rasero, Paolo Iovino, Yari Longobucco, Department of Health Sciences, University of Florence, Florence, Italy; Andrea Poliani, Ilaria Marcomini, Giulia Villa, Center for Nursing Research and Innovation, Faculty of Medicine and Surgery, Vita-Salute San Raffaele University, Milan, Italy; Alessia Campoli, Nursing Research Unit IFO, IRCCS Regina Elena National Cancer Institute, Rome, Italy

**Keywords:** cross-sectional studies, health literacy, internal–external control, parents, vaccination hesitancy, vaccines

## Abstract

**Background:**

Vaccine hesitancy remains a significant public health challenge, even in countries with high vaccination coverage, such as Italy. Understanding the sociodemographic, informational, and psychological characteristics associated with hesitancy is essential for designing effective, targeted interventions.

**Objective:**

To describe and compare the sociodemographic, behavioral, informational, and psychological profiles of vaccine-hesitant and non-hesitant Italian parents.

**Methods:**

A descriptive cross-sectional study was conducted between October 2024 and February 2025 using an anonymous online survey distributed through social media and parenting websites. Eligible participants were parents or legal guardians of children aged 0–18 years, residing in Italy. Vaccine hesitancy was assessed using the Parent Attitudes about Childhood Vaccines short form (PACV–5), alongside validated measures of health literacy (HLS-EU-Q6), vaccine literacy (HLVa–IT), adult vaccine hesitancy (aVHS), vaccine confidence (VCI), and parental health locus of control (PHLOC). Descriptive and bivariate analyses were performed to examine the differences between hesitant and nonhesitant parents.

**Results:**

Of the 308 participants, 13% were classified as vaccine-hesitant. Hesitancy was significantly associated with lower educational attainment, absence of a health care background, reliance on television and health assistants for vaccine information, lower health and vaccine literacy, lower vaccine confidence, and higher scores in the Fate subscale of the Chance domain and the Child subscale of the Internal domain of the Health Locus. A total of 89.94% of parents reported full adherence to vaccination schedules, yet hesitant parents were more likely to partially vaccinate their children and express selective vaccination intentions. Discrepancies between past vaccination behavior and future intentions were observed, suggesting that hesitancy is dynamic and potentially unstable.

**Discussion:**

Vaccine hesitancy among Italian parents is associated with low health literacy and confidence levels, distinct health beliefs, and specific information-seeking patterns. These findings suggest public health efforts should include trust-building approaches that address cognitive skills, fatalistic beliefs, and preferred communication channels. Longitudinal research is necessary to monitor changes in parental attitudes and to guide adaptive intervention strategies.

Vaccination is among the most effective public health measures, preventing 2–3 million deaths annually and leading to the eradication or sharp decline of diseases such as smallpox, polio, and measles (World Health Organization, 2025b). However, global coverage has recently decreased, with 14.3 million children left unvaccinated or undervaccinated (World Health Organization, 2025a). This decline has contributed to outbreaks of vaccine-preventable diseases, often fueled by the growing phenomenon of vaccine hesitancy (MacDonald, 2015).

Italy has reached and often exceeded 95% coverage for mandatory pediatric vaccines introduced by Law No. 119/2017, such as diphtheria–tetanus–pertussis (DTP), polio, hepatitis B, *Hemophilus influenzae* type B, and measles–mumps–rubella (MMR; Bonanni et al., 2021). In contrast, the uptake of nonmandatory vaccines, including human papillomavirus (HPV), meningococcal, and influenza vaccines, remains lower, especially among adolescents and adults, with persistent regional disparities (Cadeddu et al., 2021). Disparities across regions also persist, with some southern areas failing to reach herd immunity thresholds (Cadeddu et al., 2021).

A central factor behind disparities in vaccination coverage is vaccine hesitancy, defined by the WHO Strategic Advisory Group of Experts as “the delay in acceptance or refusal of vaccines despite the availability of vaccination services” (MacDonald, 2015). Recent studies have further elaborated on vaccine hesitancy as a dynamic continuum ranging from total acceptance to outright refusal, incorporating a psychological component of doubt or indecision (Yang et al., 2024).

Multiple surveys have shown that although parents’ overall trust in vaccination remains high, skepticism persists among specific groups, particularly regarding newer vaccines such as HPV and those recommended during adolescence (Cadeddu et al., 2021). Hesitancy was also more pronounced during COVID-19, with fluctuations linked to media narratives, perceived side effects, and lower institutional trust (Adhikari et al., 2022).

Evidence supports the multifactorial nature of vaccine hesitancy. In fact, it is shaped by several factors, including complacency (low perceived risk of disease), convenience (access to vaccination services), and confidence (trust in vaccines, health care providers, and health policies; Ryan & Malinga, 2021). Yang et al. (2024), in a meta-analysis of over 147,000 individuals across 34 observational studies, found that vaccine hesitancy significantly increased the likelihood of adverse vaccination behaviors, with hesitant individuals being about one and a half times more likely to refuse and more than twice as likely to delay vaccination. Furthermore, Wang et al. (2023) demonstrated a strong association between parental and maternal vaccine hesitancy and an increased risk of pertussis in infants and children, with unvaccinated children being more than 14 times more likely to develop the disease than their fully vaccinated peers.

Vaccine hesitancy is not uniformly distributed across the population. Previous studies showed that the prevalence of this condition differs by geographic area, vaccine type, and sociodemographic characteristics (Millat-Martínez et al., 2025). For example, in low- and middle-income countries, vaccine hesitancy is primarily influenced by access barriers, cultural norms, and political distrust, whereas in high-income contexts, it is more strongly driven by misinformation, limited health literacy, individualistic beliefs, a lower internal parental health locus of control (PHLOC), and distrust toward pharmaceutical companies or governmental institutions (Magi, Buccione et al., 2025). Hesitancy levels also vary by vaccine type, with greater acceptance of long-standing immunizations, such as MMR or DTP, and greater reluctance toward newer or nonmandatory vaccines, such as HPV, meningococcal, and pneumococcal vaccines (Facciolà et al., 2019). Younger age, lower education, and socioeconomic disadvantage, often compounded by social media exposure, further increase vulnerability to hesitancy (Singh et al., 2022).

Addressing vaccine hesitancy requires a deeper understanding of the psychological, social, and contextual factors that shape parental attitudes and behaviors toward immunization (Singh et al., 2022). While international evidence on these determinants is growing, Italian studies remain limited and often fragmented, with few analyses exploring the interplay between cognitive, emotional, and sociodemographic variables. This gap highlights the need for a descriptive study that provides a comprehensive profile of hesitant and nonhesitant parents in Italy, generating evidence to inform tailored strategies that enhance vaccine uptake and strengthen individual protection and community immunity.

The aim of this study was to identify the levels of childhood vaccine hesitancy among a sample of Italian parents and to describe the sociodemographic, behavioral, informational, and psychological factors associated with it.

## METHODS

### Study Design and Setting

In reporting this study, we adhered to the Strengthening the Reporting of Observational studies in Epidemiology checklist (Von Elm et al., 2014). Data were collected between October 28, 2024, and February 28, 2025, using an anonymous, self-administered online survey hosted on the EUSurvey platform (Koykka, 2024). The study was conducted in accordance with the principles of the Declaration of Helsinki and received ethical approval from the University of Parma Research Ethics Board (Approval No. 119-2024-N).

### Participants

Participants were recruited using snowball sampling via major social media platforms (e.g., Facebook, Instagram, and WhatsApp; Leighton et al., 2021). This strategy was adopted to efficiently reach a broad, heterogeneous group of Italian parents by leveraging peer-to-peer sharing, so that parents who received the survey could easily share it with other parents in their social circles. Eligible participants were adults aged 18 or older, self-identified as parents or legal guardians of at least one child aged 0–18, and fluent in Italian. No exclusion criteria were applied to the study. Informed consent was obtained digitally before participation. The survey was completed in a single session of ∼40  min, and no personal identifiers were collected. All questions were mandatory, which prevented missing data. However, snowball recruitment via social media may have favored participation by urban, digitally connected, predominantly female parents, potentially limiting representativeness.

### Sample Size

The sample size was determined using the standard formula for cross-sectional studies (Charan & Biswas, 2013). Based on prior estimates of parental vaccine hesitancy in Italy (proportion=23%; Napolitano et al., 2018), a 95% confidence level (z=1.96), and a 5% margin of error (*d*=.05), the minimum required sample size was calculated to be 274 participants. The target sample size was increased to 300 to enhance the representativeness of the study population and ensure sufficient variability across key sociodemographic characteristics, such as age, education, and geographical distribution.

### Data Collection and Measures

The online questionnaire was structured into two sections: the first section contained study information and informed consent, and the main questionnaire included all study variables. The EUSurvey platform was configured to ensure data integrity and completeness. The data were collected anonymously and securely stored, with access restricted to the research team only. The primary outcome of interest was parental vaccine hesitancy, assessed using the five-item short form of the Parent Attitudes about Childhood Vaccines Scale (PACV–5; Magi, El Aoufy et al., 2025). Additional constructs were measured using validated instruments, including general health literacy (HLS–EU–Q6; Lorini et al., 2019), vaccine literacy (HLVa–IT; Biasio et al., 2020), adult vaccine hesitancy (aVHS; Ledda et al., 2022), vaccine confidence (VCI; Arzilli et al., 2023), and parental health locus of control (PHLOC; Bonichini et al., 2009). All instruments were obtained with permission from the original authors before their use in this study. A complete list of instruments, scoring systems, measured constructs, and psychometric properties is presented in Table 1.

**TABLE 1:** Summary of the Measurement Instruments Used in the Study

Instrument	Target Construct	Dimensions or Subscales	Items & Format	Scoring & Interpretation	Psychometric Properties	References
PACV-5	Parental vaccine hesitancy	Unidimensional	5 items; 3-point Likert (0–2)	Total score=raw sum of 5 items (range 0–10). Higher scores=greater parental hesitancy. In this study, participants were classified as nonhesitant (<5) or hesitant (≥5)	*α*=.80; *ω*= .81	Magi, El Aoufy et al. (2025)
HLS-EU-Q6	General health literacy	Unidimensional	6 items; 4-point Likert (1–4)	Mean of at least 5 valid responses (range 1–4). Classification on the mean (nonrescaled): ≤2.50=inadequate; >2.50–2.98=problematic; >2.98–3.52=sufficient; >3.52=excellent	α=.67	Lorini, et al. (2019)
HLVa-IT	Vaccine literacy	3 subscales: Functional, Interactive, Critical	12 items; 4-point Likert (1–4)	Mean score per subscale and total (range 1–4). Higher scores=greater vaccine literacy. Suggested thresholds: <2.5 low; 2.5–2.99 medium; ≥3 high	α=.82, ICC=.48 (Functional); α=.88, ICC=.55 (Interactive); α=.90, ICC=.65 (Critical)	Biasio et al. (2020)
aVHS	General vaccine hesitancy (adults)	Unidimensional	10 items; 5-point Likert (1–5)	Final score=total sum after reverse scoring of items 5, 9, 10 (range 10–50); higher=greater adult hesitancy. Equivalent dichotomy: ≤25=low hesitancy; >25=high hesitancy	α=.94; ICC=.87	Ledda et al. (2022)
VCI	Vaccine confidence, hesitancy, and anxiety	Unidimensional	6 items selected from original 10; 5-point Likert (1–5)	Score=(L2+L3+L9)/(L4+L5+L10), range ≈0.2–5. Interpretation: <1 low confidence; ≈1 balanced; >1 high confidence	α=.82; ω=.87	Arzilli et al. (2023)
PHLOC	Health locus of control in parenting	6 subscales: Parent, Child, Fate, Divine, Media, Professional	28 items; 6-point Likert (1–6)	Mean score for each subscale (range 1–6). Higher values=stronger belief in that domain. Subscales can be grouped into Internal=Parental, Child; External—Powerful Others=Professional, Media; External—Chance=Divine, Fate	α > .70 per subscale (Parent, Child, Fate, Divine, Media, Professional)	Bonichini et al. (2009)

*Note.* aVHS=Adult Vaccine Hesitancy Scale; HLS-EU-Q6=Health Literacy Survey-Short Short Version; HLVa-IT=Health Literacy about Vaccination-Italian Version; ICC=Intraclass Correlation Coefficient; PACV-5=Parent Attitudes about Childhood Vaccines; PHLOC=parental health locus of Control; VCI= Vaccine Confidence Index; *α*=Cronbach’s alpha (internal consistency); *ω*=McDonald’s omega (internal consistency).

### Data Analysis

Data analysis was conducted using Jamovi (Version 2.4.14; https://www.jamovi.org/download.html) and Stata (Version 18.0; https://www.stata.com/stata18/). Descriptive statistics were calculated for all variables. Continuous variables were summarized as medians (MDN) and interquartile ranges (IQR), depending on distribution, assessed using the Shapiro–Wilk test. Categorical variables are reported as absolute and relative frequencies. Bivariate analyses were conducted to examine associations between parental vaccine hesitancy and sociodemographic, behavioral, informational, and psychological variables. Chi-square tests were used for comparisons involving categorical variables, while the Wilcoxon rank-sum test (Mann–Whitney *U*) was applied to continuous variables, since their distributions were not normal. Vaccination behavior was coded as “fully vaccinated” when parents reported having administered all recommended vaccines to their child, and as “partially vaccinated” when only some vaccines were administered. Based on behavior and intention, four categories were derived: “yes–yes” (vaccinated and intending to vaccinate in the future), “no–no” (not vaccinated and not intending), “yes–no” (vaccinated but not intending), and “no–yes” (not vaccinated but intending). These classifications were analyzed using McNemar’s test to assess the consistency between parents’ past vaccination behavior and their future intentions and between vaccination decisions for their first child and subsequent children. No data imputation was necessary because there was no missing data. For analyses involving subsequent children, only parents with valid paired responses for both variables in the comparison were included, resulting in small variations in sample size across tests. Because only descriptive and bivariate analyses were performed, potential confounding could not be addressed, and the results should therefore be interpreted as associative rather than causal. Given the descriptive and exploratory nature of this study, no multivariate or sensitivity analyses were conducted. This choice was also informed by the limited availability of prior Italian studies on parental vaccine hesitancy, which constrained the formulation of robust a priori hypotheses for multivariate modeling. In the analysis, parents who reported no intention to vaccinate were categorized as vaccine-hesitant.

## RESULTS

### Participant Characteristics

A detailed comparison of sociodemographic variables by hesitancy status is shown in Table 2. Overall, 308 parents participated in the study, of whom 40 (13%) were classified as vaccine-hesitant and 268 (87%) as nonhesitant, according to their stated intentions regarding future child vaccination. Most participants were female (*n*=245; 79.6%), with a median age of 43 years (IQR=37–49.5), and no significant age differences were observed between the groups. Educational attainment varied across the sample: 18.9% (*n*=58) had a high school diploma or less, 44.5% (*n*=137) held a bachelor’s degree, and 36.7% (*n*=113) had completed postgraduate education. A significant difference in the educational level was observed between hesitant and nonhesitant parents (*p*<.001). Most participants were married or partnered (*n*=262; 85.1%), and 48.7% (*n*=150) reported being health care workers, with a significantly lower hesitancy rate in this group (*p*=.004). Participants were geographically distributed across Italy, with 15% (*n*=46) residing in the north, 47.1% (*n*=145) in the center, and 39% (*n*=117) in the south or on islands. Most lived in urban areas (*n*=265; 86%), and the median number of children was two.

**TABLE 2:** Sociodemographic Characteristics of the Sample and Comparison Between Hesitant and Nonhesitant Parents (*N*=308)

	Total (*N*=308)	Hesitant (*n*=40)	Nonhesitant (*n*=268)	*p*
Gender (female), *n* (%)	245 (79.6)	31 (12.7)	214 (87.6)	.731
Age, *MDN* (IQR)	43 (37–49.5)	42 (37–48.5)	43(37–50)	.680
Education, *n* (%)				<.001
** **High school or lower	58 (18.8)	18 (31)	40 (69)	
** **Bachelor’s degree	137 (44.5)	16 (11.7)	121 (88.3)	
** **Postgraduate degree	113 (36.7)	6 (5.3)	107 (94.7)	
Marital status, *n* (%)				.414
** **Married or partnered	262 (85.1)	31 (11.8)	231 (88.2)	
** **Divorced	27 (8.8)	6 (22.2)	21 (77.8)	
** **Single	15 (4.9)	2 (13.3)	13 (86.7)	
** **Other	4 (1.3)	1 (25.)	3 (75)	
Health care worker, *n* (%)	150 (48.7)	11 (7.3)	139 (92.7)	.004
Region, n (%)				.884
** **North Italy	46 (14.9)	7 (15.2)	39 (84.8)	
** **Central Italy	145 (47.1)	18 (12.4)	127 (87.6)	
** **South and Islands	117 (38)	15 (12.8)	102 (87.2)	
Living area, *n* (%)				.489
** **City	265 (86)	33 (12.4)	232 (87.6)	
** **Rural	43 (14)	7 (16.3)	36 (83.7)	
Number of children, MDN (IQR)	2 (1–2)	2 (1–2)	2 (1–2)	.669
Source of Information, *n* (%)^a^				
** **General practitioner	267 (86.7)	35 (13.1)	232 (86.9)	.871
** **Health institutions	165 (53.8)	19 (11.5)	146 (88.5)	.409
** **Scientific databases	81 (26.3)	5 (6.2)	76 (93.8)	.034
** **Internet	61 (19.8)	10 (16.4)	51 (83.6)	.377
** **Nurse	39 (12.7)	6 (15.4)	33 (84.6)	.634
** **Social media	35 (11.4)	7 (20)	28 (80)	.190
** **Friends or relatives	24 (7.8)	4 (16.7)	20 (83.3)	.577
** **Community health worker	20 (6.5)	6 (30)	14 (70)	.019
** **Magazines	20 (6.5)	5 (25)	15 (75)	.098
** **Television	19 (6.2)	6 (31.6)	13 (68.4)	.013

*Note*. IQR=interquartile range; MDN=median; *N*=total sample; *n*= subgroup size; *p*=probability value.

Data are presented as *N* (%) for categorical variables and as MDN (IQR) for continuous variables. *p*values are from chi-square or Mann–Whitney *U* tests.

^a^ Participants could select multiple sources and percentages refer to the proportion of parents in each hesitancy group who reported using each source.

### Patterns of Vaccination Behavior and Future Intentions

Among all parents, 90% (*n*=277) reported having administered all vaccinations to their first child, and 10.1% (*n*=31) reported partial vaccination (i.e., their child had received only some of the recommended vaccines). No parent reported total nonvaccination. Vaccine hesitancy was more prevalent among those who had partially vaccinated their first child (*n*=15; 48.4%) than among those who had fully vaccinated them (*n*=25; 9%). Among parents with more than one child (*n*=177; 57.5%), similar patterns were observed: 93% (*n*=160) reported full vaccination of their subsequent children. Regarding intentions, 83% (*n*=256) of parents intended to administer all recommended vaccinations to their first child, 15.6% (*n*=48) intended to vaccinate selectively, and 1.3% (*n*=4) expressed no intention to vaccinate. These patterns were replicated in the parents’ intentions regarding subsequent children. Statistically significant differences between hesitant and nonhesitant parents were observed for all measures of vaccination behavior and intention (all *p* <.001). The full data are presented in Table 3.

**TABLE 3:** Vaccination Behaviors and Intentions According to Parental Hesitancy Status (*N*=308)

	Total (*N*=308)	Hesitant (*n*=40)	Nonhesitant (*n*=268)	*p*
Vaccination of first child, *n* (%)				<.001
Fully vaccinated	277 (89.9)	25 (9)	252 (91)	
Partially vaccinated	31 (10.1)	15 (48.4)	16 (51.6)	
Not vaccinated	0 (0.0)	0 (0.0)	0 (0.0)	
Vaccination of subsequent children, *n* (%)				<.001
Fully vaccinated	160 (93)	15 (9.4)	145 (90.6)	
Partially vaccinated	12 (7)	7 (58.3)	5 (41.7)	
Not vaccinated	0 (0.0)	0 (0.0)	0 (0.0)	
Intention to vaccinate first child, *n* (%)				<.001
Fully vaccinated	256 (83.1)	16 (6.3)	240 (93.8)	
Partially vaccinated	48 (15.6)	20 (41.7)	28 (58.3)	
Not vaccinated	4 (1.3)	4 (100)	0 (0.0)	
Intention to vaccinate other children, *n* (%)				<.001
Fully vaccinated	144 (83.7)	11 (7.6)	133 (92.4)	
Partially vaccinated	26 (15.1)	10 (38.5)	16 (61.5)	
Not vaccinated	2 (1.2)	1 (50)	1 (50)	

*Note.* *n*=subgroup size; *N*=total sample; *p*=probability value.

Values are reported as *n* (%). Percentages refer to the proportion of parents within each hesitancy group. *p-*values are from chi-square tests.

### Health Information Sources and Parental Vaccine Hesitancy

The general practitioner was the most frequently cited source of information about childhood vaccination (*n*=267, 86.7%), followed by health institutions (*n*=165, 53.6%) and scientific databases (*n*=81, 26.3%). The use of other sources, such as the internet, nurses, social media, and community health workers, was less common. Hesitant parents more frequently reported relying on health assistants (*n*=6; 30%) and television (*n*=6; 31.9%), both significantly more than their nonhesitant counterparts (*p*=.019 and *p*=.013, respectively). Conversely, the use of scientific databases was significantly more frequent among nonhesitant parents (*p*=.034). No significant group differences were observed for the remaining sources. The findings are summarized in Table 1.

### Psychological Measures

Hesitant participants reported significantly lower health literacy levels, as indicated by median scores on the HLS–EU–Q6 (*MDN*=2.2, IQR=1.8–2.7) compared with their nonhesitant counterparts (*MDN*=2.5, IQR=2.2–3; *p*=.004). Similarly, vaccine-related health literacy, measured by the HLVa–IT, was lower among hesitant parents (*MDN*=3.2, IQR=2.7–3.6) than among nonhesitant parents (*MDN*=3.5, IQR=3.1–3.7; *p*=.007). Regarding vaccine confidence, hesitant parents scored significantly lower on the VCI (MDN=1, IQR=0.67–1.2) than nonhesitant parents (*MDN*=2.2, IQR=1.7–3; *p* <.001). This trend was confirmed by the aVHS, in which hesitant participants reported lower vaccine hesitancy scores (*MDN*=30.5, IQR=26–36) than the nonhesitant group (MDN=44, IQR=39–48; *p* <.001). Regarding parental PHLOC, significant differences between hesitant and nonhesitant parents emerged in two domains. Within the PHLOC internal dimension, hesitant parents reported higher scores on the child subscale (*MDN*=2.9, IQR=1.9–3.5 vs. *MDN*=2.3, IQR=1.8–3.2; *p*=.045), indicating a greater attribution of control to the child, whereas no significant difference was observed on the parent subscale (*p*=.124). Within the PHLOC external-chance dimension, hesitant parents showed higher scores on the Fate subscale (*MDN*=2, IQR=1.2–2.8 vs. *M* *DN*=1.3, IQR=0.83–2; *p*=.008), whereas no difference was found on the divine subscale (*p*=.377). No significant differences were detected in the PHLOC external-powerful others dimension, including the professional (*p*=.118) and media (*p*=.161) subscales. A borderline result was observed for the external-chance subscale (*p*=.054), with hesitant parents reporting slightly higher scores. Full descriptive statistics for all psychological measures are presented in Table 4.

**TABLE 4:** Psychological Measures by Hesitancy Group (*N*=308)

	Total (*N*=308)	Hesitant (*n*=40)	Nonhesitant (*n*=268)	*p*
HLS-EU-Q6	2.5 (2.2–3)	2.2 (1.8–2.7)	2.5 (2.2–3)	.004
HLVa-IT	3.5 (3.1–3.7)	3.2 (2.7–3.6)	3.5 (3.1–3.7)	.007
VCI	2 (1.4–3)	1 (0.67–1.2)	2.2 (1.7–3)	<.001
aVHS	43 (38–47)	30.5 (26–36)	44 (39–48)	<.001
PHLOC Parent	3.9 (3.3–4.4)	3.8 (3.1–4.3)	3.9 (3.3–4.5)	.124
PHLOC Fate	1.4 (0.92–2.2)	2 (1.2–2.8)	1.3 (0.83–2)	.008
PHLOC Divine	0.75 (0.75–1.8)	0.87 (0.75–1.9)	0.75 (0.75–1.8)	.377
PHLOC Child	2.5 (1.8–3.2)	2.9 (1.9–3.5)	2.3 (1.8–3.2)	.045
PHLOC Professional	4 (3.4–4.8)	3.7 (2.8–4.8)	4 (3.4–4.8)	.118
PHLOC Media	3.3 (2–4.3)	3.7 (2.7–4.3)	3.2 (2–4.3)	.161
PHLOC Internal	6.3 (5.4–7.3)	6.8 (5.3–7.5)	6.3 (5.4–7.3)	.681
PHLOC External-powerful others	7.3 (6–8.6)	7.2 (5.5–9.1)	7.3 (6–8.5)	.962
PHLOC External-chance	3.8 (1.8–3.8)	3.3 (2–4.2)	2.4 (1.8–3.6)	.054

*Note*. aVHS=Adult Vaccine Hesitancy Scale; HLS-EU-Q6=European Health Literacy Survey Questionnaire (short form); HLVa-IT=Health Literacy Vaccinale per Adulti; *n*=subgroup size; *N*=total sample; *p*=probability value; PHLOC=parental health locus of control; VCI=Vaccine Confidence Index.

Data are reported as median (interquartile range) for skewed variables. *p-*values are from Mann–Whitney *U* tests.

### Concordance Between Intention and Behavior

Among all parents, 247 (80.2%) reported having fully vaccinated their first child and stated that they would do so again in the future (“yes–yes”), while 22 (7.1%) had neither vaccinated nor intended to (“no–no”). In contrast, 30 (9.7%) parents reported having vaccinated their first child but did not intend to do so in the future (“yes–no”), and nine (2.9%) had not vaccinated but expressed future intention (“no–yes”). This inconsistency was statistically significant (*χ^2^* =11.3, *p* <.001). Among parents with more than one child (*n*=177; 57.5%), 171 (55.5%) provided complete data for the analysis comparing vaccination behavior and intention for subsequent children. Of these, 142 (46.1%) had fully vaccinated their subsequent children and intended to do so again, while 11 (3.6%) were consistent in not vaccinating and not intending to do so. Seventeen parents (5.5%) had vaccinated their children but did not intend to continue, and one reported the opposite pattern. This inconsistency was also significant (*χ^2^* =14.2, *p* <.001). Finally, actual vaccination behavior was compared between first and subsequent children among parents with complete data (*n*=172; 55.8%). Among them, 155 (50.3%) parents reported having fully vaccinated both children, and 12 (3.9%) had vaccinated neither child. Five parents (1.6%) vaccinated only their first child, and none reported vaccinating only their subsequent children. This discordance was also statistically significant (*χ^2^* =5, *p*=.025). The data are presented in Table 5.

**TABLE 5:** Concordance Between Past Vaccination Behavior and Future Intention, and Between Children, Assessed With McNemar Test (*N*=308)

Comparison	Consistent Behavior	Inconsistent Behavior	Total (*N*)	*χ^2^*	***p***
First child: vaccination vs. intention	247 (yes–yes), 22 (no–no)	30 (yes–no), 9 (no–yes)	308	11.3	<.001
Second child: vaccination vs. intention	142 (yes–yes), 11 (no–no)	17 (yes–no), 1 (no–yes)	171	14.2	<.001
First vs. second child: actual vaccination	155 (both vaccinated), 12 (both unvaccinated)	5 (only first vaccinated), 0 (only second vaccinated)	172	5	.025

*Note. N*=total sample; *p*=probability value; *χ^2^* =chi-square statistic.

Values refer to matched comparisons between reported behavior and stated intentions. “yes–yes”=vaccinated and intended to vaccinate; “no–no”=not vaccinated and did not intend to vaccinate; “yes–no”=vaccinated but did not intend to; “no–yes”=did not vaccinate despite intention. For analyses involving subsequent children, only parents with valid paired responses for both variables were included, which explains the lower *n* compared with the total number of parents with ≥2 children. *p*values are from McNemar’s test.

## DISCUSSION

We explored the sociodemographic, informational, and psychological factors associated with vaccine hesitancy among Italian parents. Our findings revealed that hesitancy was more prevalent among those with lower educational attainment and no health care background and was associated with partial vaccination of children and reduced intention to vaccinate in the future. Hesitant parents relied more on informal sources such as television and health assistants and demonstrated lower levels of health and vaccine literacy, lower confidence in vaccines, and a more external health locus of control, particularly related to fate and the perceived autonomy of the child. Notably, this study highlights discrepancies between past vaccination behaviors and future intentions and offers novel insights into the psychological underpinnings of parental decision-making, especially regarding the locus-of-control dimensions that are rarely investigated in this context.

Consistent with the existing literature, lower education levels and the absence of a health care background were associated with greater vaccine hesitancy (Lamot & Kirbiš, 2024). These results support prior research suggesting that limited access to reliable health information and reduced scientific trust may contribute to hesitancy, particularly among less educated groups (Cagnotta et al., 2025). Working in health care settings appears to have a protective effect, as evidenced by lower hesitancy rates among health care workers, as shown in the literature (Elizondo-Alzola et al., 2021), which likely reflects major exposure to medical knowledge and perceived disease risk. No significant gender differences emerged in our results, in line with recent evidence showing that parental gender is not a consistent predictor of vaccine hesitancy but rather interacts with broader sociodemographic and contextual factors (Cagnotta et al., 2025). Although most participants were from central Italy and urban areas, no regional or urban-related differences in hesitancy were observed. This finding contrasts with recent evidence of substantial disparities in vaccination coverage across Italian regions (Signorelli et al., 2025). A likely explanation is that our study focused on parental intentions rather than actual coverage, used an online self-selected sample that may have attenuated regional variability, and included relatively small subgroups of participants. The relatively low prevalence of hesitant parents in our sample may also reflect the effect of Italy’s current mandatory childhood vaccination policy, particularly for school-aged children.

Vaccination behaviors and intentions were generally favorable, with most parents reporting full adherence to childhood immunization schedules and expressing their intention to vaccinate in the future. Importantly, intention and behavior were highly consistent, particularly among nonhesitant parents, reinforcing the utility of intention-based measures in research on vaccine hesitancy. This alignment is echoed by prospective evidence showing that parental intention—along with attitudinal and normative components—predicts actual child vaccination uptake (Lau et al., 2023). Broad reviews corroborate that attitude is the strongest predictor of intention, followed by subjective norms, while the findings for perceived behavioral control are mixed (Gentile & Alesi, 2024). However, the relationship between intention and behavior is not always straightforward, as other studies have indicated (Obohwemu and Christie-de Jong, 2022). The stronger coherence observed in our sample may reflect the timing of the measurement (post-behavior), the selected nature of the online sample, and the reinforcing effect of Italy’s mandatory vaccination policy.

Information sources differed significantly by hesitancy level. General practitioners were the most trusted source across the sample, confirming their central role in guiding parental vaccine decisions (Shen & Dubey, 2019). However, as our analyses do not allow causal inferences, the observed association may also reflect that parents with fewer hesitations are more likely to consult their general practitioners, rather than consultation itself directly reducing hesitancy. Hesitant parents were more likely to report reliance on less specialized or evidence-based channels, such as television or informal media, which were associated with higher hesitancy levels in our sample (Eller et al., 2019). The media’s influence on vaccine uptake is largely mediated by hesitancy, with television playing an especially inconsistent role (Recio-Román et al., 2023). Conversely, nonhesitant parents more often reported using scientific databases and professional health information, consistent with higher digital and vaccine literacies (Ashfield et al., 2024). These results highlight the need to equip a broad range of health professionals, including school and family nurses as well as physicians, with accurate communication tools (Wilson et al., 2023) and to tailor outreach strategies to the media channels through which hesitant parents are most often reached.

Psychological constructs strongly differentiated hesitant from nonhesitant parents. Lower health and vaccine literacy scores were observed among hesitant parents, confirming past findings that underscore the importance of cognitive skills in vaccine-related decisions (Martinelli & Veltri, 2021). Measures of vaccine confidence and adulthood hesitancy also clearly discriminated between groups, with hesitant parents reporting lower confidence and higher concern, consistent with evidence that mistrust and perceived risks are central determinants of hesitancy (Caudal et al., 2020). Regarding PHLOC, hesitant parents scored significantly higher on the fate and child subscales, suggesting a more fatalistic orientation and stronger attribution of health outcomes to the child’s behavior rather than parental influence. This pattern echoes previous work showing that external or fatalistic orientations are associated with lower adherence to preventive behavior (Shen, 2017). No significant differences emerged across other subscales, suggesting that the relationship between PHLOC and vaccine attitudes is more context-dependent. However, given the cross-sectional design, these associations cannot establish directionality or causation, and the interpretation of PHLOC patterns should therefore be considered exploratory. Taken together, these findings suggest that hesitant parents may endorse both greater external attributions (e.g., fate, child responsibility) and weaker internal parental control, which may undermine their perceived agency in making vaccination-related decisions. The literature reports that a weaker internal PHLOC is associated with lower adherence to vaccination recommendations (Magi, Buccione et al., 2025).

Finally, the discrepancies observed between past vaccination behavior and future intentions—particularly among parents who had previously vaccinated but no longer intended to—suggest that hesitancy is a dynamic rather than a fixed phenomenon. Similar findings have been reported in qualitative studies, where parenthood and evolving social contexts have reshaped vaccine attitudes over time (Rozbroj et al., 2020). Changes in trust in health care providers, exposure to conflicting media messages, and shifting perceptions of vaccine risks have all been identified as factors contributing to these fluctuations.

The findings of this study have several practical and research implications. Clinically, identifying specific sociodemographic and psychological profiles associated with vaccine hesitancy underscores the need for tailored communication strategies. Health care professionals—particularly general practitioners and other frontline providers—should be trained to recognize signs of hesitancy and adapt their messaging to parents’ educational level, occupational context, information sources, and health beliefs. Interventions should not only enhance general and vaccine-specific literacy but also address fatalistic beliefs and trust deficits through empathetic, evidence-based dialogue. For example, pediatric counseling during routine visits, school-based educational initiatives, and informational materials tailored to different literacy levels may all help strengthen parents’ vaccine confidence.

From a research standpoint, the results underscore the importance of longitudinal studies in monitoring changes in parental vaccine attitudes over time and evaluating the stability of hesitancy within families. The observed behavioral-intentional inconsistencies suggest that vaccine decisions are dynamic rather than fixed, requiring further investigation into the factors that predict reversals in intention or behavior. Future research employing random or stratified sampling methods would improve representativeness and allow for stronger generalization of findings across diverse parental populations. Moreover, validating measurement tools in the Italian context—particularly those lacking established clinical thresholds—would improve the precision of future studies and support their integration into public health-monitoring systems. In addition, interventional studies testing tailored communication strategies and family-centered approaches would be valuable for identifying effective methods for reducing hesitancy and strengthening vaccine confidence. In addition, interventional studies on tailored and family-centered communication are needed to reduce hesitancy and build confidence, while qualitative research could explore parental motivations in depth, and quantitative studies could assess the effectiveness of educational strategies on outcomes such as uptake, literacy, and trust. Overall, this study provides original and comprehensive evidence of the sociodemographic, psychological, and behavioral characteristics of vaccine-hesitant Italian parents. Effective public health strategies should move beyond purely informational approaches by integrating cognitive, attitudinal, and contextual dimensions into targeted interventions that explicitly consider education, occupation, trust, and beliefs regarding health control. Such tailored strategies may strengthen communication and engagement, ultimately enhancing the effectiveness and sustainability of vaccination programs in the future.

### Strengths and Limitations

This study has two main strengths. First, the use of validated instruments allowed for a reliable assessment of general and vaccine-specific psychological constructs. Most instruments had been previously validated in Italian or adapted following standardized procedures to ensure linguistic and cultural appropriateness. Second, the inclusion of behavioral measures (actual vaccination behavior) and attitudinal indicators (future intentions) provided a multidimensional characterization of vaccine hesitancy that aligned with contemporary conceptual frameworks.

This study had some limitations. The use of snowball sampling likely introduced self-selection bias, favoring digitally connected and motivated parents and leading to an overrepresentation of urban residents and health care workers, which may limit generalizability. The cross-sectional design precluded causal inference, and the brief PACV–5 may not have fully captured the spectrum of hesitancy. In addition, the lack of multivariate analyses limited our ability to adjust for potential confounders. Moreover, as data were self-reported, the accuracy of responses may have been affected by recall or social desirability bias, despite the survey’s anonymity. The requirement of fluency in Italian may also have excluded parents from migrant or linguistic minority groups, further limiting representativeness. Finally, subgroup analyses (e.g., by geographical area) may have been underpowered, and the sample was predominantly female and urban, which may further limit the generalizability of the findings.

## CONCLUSION

The results of this study provide one of the first comprehensive profiles of vaccine-hesitant and nonhesitant Italian parents. Vaccine hesitancy was linked to lower levels of education, limited health and vaccine literacy, reduced confidence, and external or fatalistic health beliefs. Despite generally high vaccination uptake, intrafamily and interfamily discrepancies highlight the dynamic nature of parental attitudes toward vaccinating their children. Addressing hesitancy requires context-sensitive strategies that go beyond information. Researchers should examine how parental profiles evolve and assess tailored interventions to reinforce vaccine confidence.
